# Surgical publication activity in the English literature over a 10‐year interval

**DOI:** 10.1002/bjs5.50172

**Published:** 2019-05-29

**Authors:** D. L. Hinrichs, E. S. Debus, R. T. Grundmann

**Affiliations:** ^1^ Department of Vascular Medicine University Heart Centre, University Hospital Hamburg‐Eppendorf 52 Martinistrasse 20246 Hamburg Germany

## Abstract

**Background:**

Surgical publication activity in the English literature over a 10‐year interval may have changed. This study sought to identify which countries make the most contributions and whether significant shifts have occurred in this time.

**Methods:**

Screening of 17 international journals in PubMed was performed for the time periods 2006–2007 and 2016–2017, for papers published by a first author belonging to a general surgical department. Data were collected by country regarding the total number of publications, cumulative impact factors (IFs), publications per inhabitant, IFs per inhabitant, and number of RCTs, meta‐analyses and systematic reviews per country in both periods.

**Results:**

A total of 2247 and 3029 papers were found for 2006–2007 and 2016–2017 respectively. In 2006–2007, most papers (605, 26·9 per cent; 2697·3 IFs) came from the USA, followed by Japan (284, 12·6 per cent; 1042·1 IFs) and the UK (197, 8·8 per cent; 923·1 IFs). In 2016–2017, the USA led again with 898 papers (29·6 per cent; 4575·3 IFs), followed by Japan with 414 papers (13·7 per cent; 1556·6 IFs) and the Netherlands with 167 (5·5 per cent; 885·2 IFs). From the top 15 countries, Sweden, the Netherlands and Switzerland contributed the most articles per inhabitant during both time periods. During both periods, the UK published the most RCTs, meta‐analyses and systematic reviews.

**Conclusion:**

Surgeons from the USA were the most productive in total number of publications during both time periods. However, smaller European countries were more active than the USA in relation to their population size.

## Introduction

Publication of surgical research is important. Understanding trends in sources and volumes of published material provides some information about changes in levels of research activity and quality. The present study looked at publication activity in clinical general surgery by screening a sample of English‐language journals over an interval of 10 years. The aims of this analysis were to check which countries published the most articles in these journals and to compare their activity in both time periods.

The cumulative total of impact factors (IFs) was analysed per country, reflecting not only the number of articles published, on the basis that IFs may be some indication of publication quality and scientific contribution.

These analyses identified which countries contributed the greatest scientific volume and where the most important articles were generated. Trends in publication volume over the last 10 years provide some indication of the state of clinical research in general surgery.

## Methods

Between 10 December 2017 and 1 June 2018, a bibliometric analysis was performed in PubMed^1^ for the years 2006–2007 and 2016–2017. A selection of 17 international journals that focused on the field of general surgery or dealt with high‐level interdisciplinary medical topics was examined. The field of general surgery was defined as including the fields of visceral surgery (including surgery of the oesophagus, stomach, small intestine, colon, rectum, surgery of the appendix and hepatopancreatobiliary surgery), adrenal surgery, hernia surgery, obesity surgery, parathyroid/thyroid surgery and transplantation surgery (intestinal, kidney, liver and pancreas).

The selection of journals was based on the following characteristics: journals should cover a broad range of general surgical topics, a 5‐year IF for 2007 and 2017 should be available, and editorial boards should originate from different countries. The origins of various editorial boards were determined by analysing the location of their respective editorial offices. Given the large amount of data research, the decision was taken to include the same number of journals as van Rossum and colleagues^2^ (15 journals), whose paper represents the only comparable analysis. In addition, two well known high‐impact journals, the *New England Journal of Medicine* and *The Lancet*, were included because they publish prospective RCTs from all disciplines of medicine.

Inclusion of articles was based on the following criteria: the first author was part of a general surgery department, as stated clearly in the author information section; if the description of the department was inconclusive, the abstract and title had to pertain to a general surgery topic, to ensure that the author was most likely a surgeon. Furthermore, the articles had to provide an abstract on PubMed, be published in English and consist of clinical studies with a study population of at least ten adults of whom at least 75 per cent should have been 18 years or older. The article had to be published as a printed version of the journal. If the first author was part of numerous surgical departments from different countries, the article was appointed to the first surgery department listed.

Articles were excluded if they were published in the fields of breast surgery, burn wound, lung, melanoma, trauma or urogenital tract surgery, given that these fields are treated by varying specialties in different countries. Additionally, if authors published within the gynaecological, urological, orthopaedic, ear nose and throat, neurosurgery, paediatric, plastic or vascular surgery departments, the articles were excluded. In addition, studies were excluded if more than 25 per cent of the patients were younger than 18 years. Given that this study was interested in only clinical studies, non‐clinical studies, including animal studies, experimental studies without a surgical procedure, *ex vivo* models, and educational studies that omitted surgical residents or specialists were excluded. All electronic publications (Epubs) and duplicates were excluded to generate an accurate yearly count.

All articles were screened independently by two authors, by manually checking the title, author information and abstract in PubMed. To avoid interobserver and intraobserver variability, inclusion and exclusion criteria were defined clearly beforehand. All articles were screened by the same two authors; in case of doubt, a decision was based on group consensus.

For each paper the IF of the respective journal was determined, and not the number of citations for each article. For 2006–2007 publications the 5‐year IF of the year 2007 was used, and for 2016–2017 publications the 5‐year IF of the year 2017 was employed, provided by InCites Journal Citation Reports of Clarivate Analytics (2018)^3^.

The total number of publications per first author's country was counted. The cumulative sum of the IFs of the publications concerned was calculated by multiplying the number of articles by the 5‐year IF of the respective journal. The total number of papers and the cumulative sum of the IFs was divided by the number of the country's inhabitants (population of 2007 and 2017 respectively) to obtain a publication and IF per inhabitant ratio^3^, ^4^, ^5^.

Subcategorization of all RCTs, meta‐analyses and systematic reviews was performed per country, and countries were ranked according to the number of papers in this collective category.

## Results

**Figure 1:**
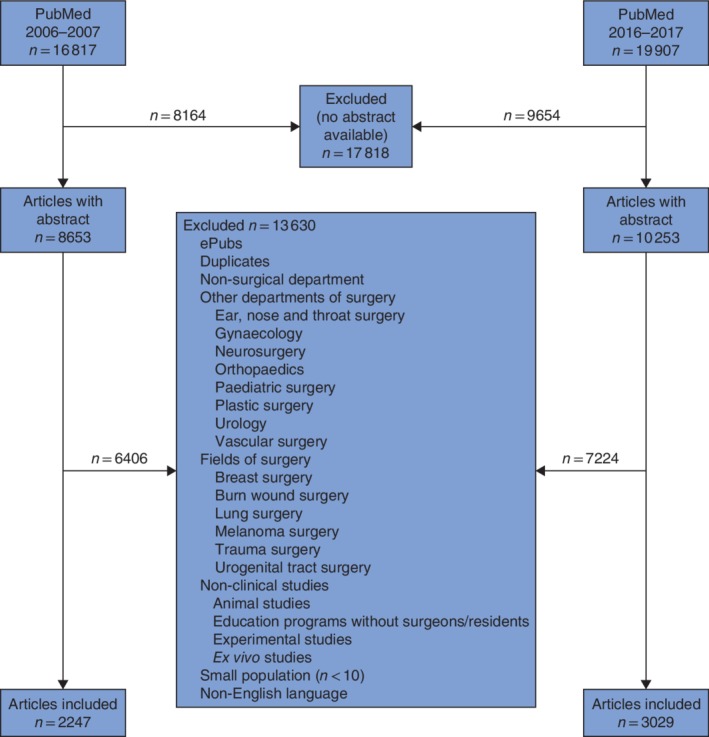
Flow diagram for the study

After screening for the availability of an abstract, 8653 articles for 2006–2007 and 10 253 for 2016–2017 remained (*Fig*. 1). After application of the inclusion and exclusion criteria, 2247 articles for 2006–2007 and 3029 for 2016–2017 remained.

*Table* 1 lists the analysed journals according to IF values in 2006–2007 compared with 2016–2017. Most journals had a higher IF in 2016–2017 than in 2006–2007. Most of the included journals had their editorial offices in the USA (12), followed by the UK (3) , Germany (1) and Switzerland (1).

**Table 1:** Analysed journals ranked by total 5‐year impact factors, 2007 and 2017

	2006–2007		2016–2017		
Rank	Journal*	5‐year IF 2007†	Journal*	5‐year IF 2017†	Increase in 5‐year IFs, 2007 *versus* 2017† (%)
1	*N Engl J Med*	45·941	*N Engl J Med*	67·512	43·2
2	*Lancet*	24·201	*Lancet*	52·665	117·6
3	*J Clin Oncol*	13·753	*J Clin Oncol*	21·455	56·0
4	*Ann Surg*	8·464	*Gut*	15·910	91·3
5	*Gut*	8·319	*Ann Surg*	9·097	7·5
6	*Oncologist*	5·193	*Br J Surg*	6·051	31·5
7	*Br J Surg*	4·602	*Thyroid*	5·769	118·9
8	*Ann Surg Oncol*	4·229	*Oncologist*	5·510	6·1
9	*Transplantation*	3·527	*J Am Coll Surg*	4·972	64·0
10	*Obes Surg*	3·134	*Ann Surg Oncol*	4·141	−2·1
11	*Dis Colon Rectum*	3·133	*Obes Surg*	4·027	28·5
12	*Surgery*	3·099	*Dis Colon Rectum*	3·947	26·0
13	*J Am Coll Surg*	3·032	*Surgery*	3·801	22·7
14	*Thyroid*	2·635	*Transplantation*	3·786	7·3
15	*World J Surg*	2·273	*World J Surg*	3·052	34·3
16	*Langenbecks Arch Surg*	1·829	*Langenbecks Arch Surg*	2·291	25·3
17	*Dig Surg*	1·559	*Dig Surg*	2·098	34·6

* Abbreviations from the US National Library of Medicine catalogue^6^;

† provided by *InCites Journal Citation Reports* (2018)^3^. IF, impact factor.

### Ranking by total number of publications and cumulative impact factors

#### *2006–2007*

The ranking of the top 15 countries in 2006–2007, based on the total number of papers published, is shown in *Table* 2. Some 2247 papers were included in this analysis. Authors from the USA were the most productive, with the highest total number of papers. The next position was occupied by Japan, followed by the UK, Germany and Italy. These top five countries combined published 1369 papers (60·9 per cent), more than half of all publications analysed for that time period. Cumulative IFs are also listed in *Table* 2. Compared with the number of publications, the USA, Japan and the UK again occupied the top three positions. Italy switched its position to fourth, whereas Germany dropped to fifth place.

**Table 2:** Top 15 countries ranked by total number of papers, 2006–2007 and 2016–2017

	2006–2007			2016–2017		
Rank	Country*	No. of papers	CIFs	Country*	No. of papers	CIFs
1	US	605 (26·9)	2697·3	US	898 (29·6)	4575·3
2	JP	284 (12·6)	1042·1	JP	414 (13·7)	1556·6
3	GB	197 (8·8)	923·1	NL	167 (5·5)	885·2
4	DE	152 (6·8)	595·5	GB	157 (5·2)	972·7
5	IT	131 (5·8)	597·0	FR	157 (5·2)	761·0
6	NL	103 (4·6)	399·2	DE	154 (5·1)	700·0
7	FR	87 (3·9)	432·5	CN	142 (4·7)	646·0
8	CN	68 (3·0)	305·8	IT	121 (4·0)	530·8
9	SE	61 (2·7)	314·6	KR	110 (3·6)	495·4
10	AU	55 (2·4)	211·3	SE	80 (2·6)	419·6
11	CA	49 (2·2)	259·3	CA	67 (2·2)	321·0
12	TW	49 (2·2)	183·7	CH	64 (2·1)	268·2
13	KR	46 (2·0)	161·9	AU	55 (1·8)	242·4
14	CH	44 (2·0)	197·5	ES	48 (1·6)	182·8
15	TR	37 (1·6)	92·3	DK	37 (1·2)	162·1
	Other (*n* = 39)	279 (12·4)	894·5	Other (*n* = 42)	358 (11·8)	1541·1
	All (*n* = 54)	2247 (100)	9307·6	All (*n* = 57)	3029 (100)	14 260·2

Values in parentheses are percentages.

* Abbreviations from ISO Country Codes for Selected Countries^7^: US, USA; JP, Japan; GB, UK; NL, the Netherlands; DE, Germany; IT, Italy; FR, France; CN, China; SE, Sweden; KR, Republic of Korea; AU, Australia; CA, Canada; TW, Taiwan; CH, Switzerland; ES, Spain; TR, Turkey; DK, Denmark. CIFs, cumulative impact factors.

In addition to the top 15 countries shown in *Table* 2, 39 other countries contributed 279 papers, representing 12·4 per cent of all papers published and providing 894·5 (9·6 per cent) of the cumulative IFs from all 54 countries.

Regarding mean IFs per publication, the UK had the highest score (4·7 IFs/publication), followed by Italy (4·6 IFs/publication), the USA (4·5 IFs/publication), Germany (3·9 IFs/publication) and Japan (3·7 IFs/publication).

#### *2016–2017*

Compared with 2006–2007, more papers (3029) were published in 2016–2017, representing an increase of 782 publications (34·8 per cent) (*Table* 2). The USA and Japan once again achieved the highest ranking. For the other positions there were significant shifts. Third place was occupied by the Netherlands, followed by the UK and France, both with the same number of papers, for which the UK had higher cumulative IFs. These five countries contributed 1793 papers in total, again more than half of all papers in this period. Germany and Italy no longer belonged in the top five rankings. The ranking order for other countries also changed (*Table* 2). In terms of cumulative IFs, the top three countries were the same as in 2006–2007: the USA was again followed by Japan, the UK, the Netherlands and France. Changes for other countries are shown in *Table* 2.

In addition to the top 15 countries shown in *Table* 2, a total of 42 countries published 358 papers, representing 11·8 per cent of all papers published and 1541·1 (10·8 per cent) of the total cumulative IFs from all countries together.

**Figure 2:**
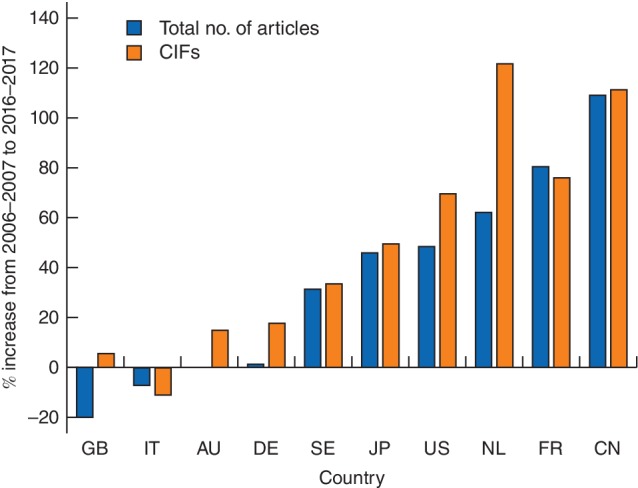
Increase in total number of articles and cumulative impact factors between 2006–2007 and 2016–2017. Values are shown for the top ten countries in 2006–2007. Abbreviations from ISO Country Codes for Selected Countries^7^: GB, UK; IT, Italy; AU, Australia; DE, Germany; SE, Sweden; JP, Japan; US, USA; NL, the Netherlands; FR, France; CN, China. CIFs, cumulative impact factors

In 2016–2017, most of the former top ten countries achieved higher cumulative IFs than in 2006–2007 (*Fig*. 2).

Regarding mean IFs per publication, the UK again ranked first (6·2 IFs/publication), followed by the Netherlands (5·3 IFs/publication), the USA (5·1 IFs/publication), France (4·8 IFs/publication) and Japan (3·8 IFs/publication).

### Number of publications and impact factors based on population

The number of publications per million inhabitants and cumulative IFs are given in *Table* 3. Sweden, the Netherlands and Switzerland were the top three in both 2006–2007 and 2016–2017, with Sweden and the Netherlands exchanging positions.

**Table 3:** Top 15 countries ranked by total number of publications per inhabitant, 2006–2007 and 2016–2017

	2006–2007			2016–2017		
Rank	Country*	Publications per 10^6^ inhabitants	Impact factor per 10^6^ inhabitants	Country*	Publications per 10^6^ inhabitants	Impact factor per 10^6^ inhabitants
1	SE	6·66	34·4	NL	9·74	51·7
2	NL	6·28	24·4	SE	7·94	41·7
3	CH	5·82	26·2	CH	7·55	31·7
4	GB	3·21	15·1	DK	6·41	28·1
5	AU	2·64	10·1	JP	3·26	12·3
6	IT	2·24	10·2	US	2·75	14·1
7	JP	2·21	8·1	GB	2·37	14·7
8	TW	2·13	8·0	FR	2·33	11·3
9	US	2·00	9·0	AU	2·23	9·9
10	DE	1·84	7·2	KR	2·13	9·6
11	CA	1·48	7·9	IT	1·99	8·8
12	FR	1·35	6·8	DE	1·86	8·5
13	KR	0·94	3·3	CA	1·82	8·7
14	TR	0·53	1·3	ES	1·03	3·9
15	CN	0·05	0·2	CN	0·10	0·5

* Abbreviations from ISO Country Codes for Selected Countries^7^: SE, Sweden; NL, the Netherlands; CH, Switzerland; GB, UK; DK, Denmark; AU, Australia; JP, Japan; IT, Italy; US, USA; TW, Taiwan; FR, France; DE, Germany; KR, Republic of Korea; CA, Canada; TR, Turkey; ES, Spain; CN, China.

### Ranking by number of RCTs, meta‐analyses and systematic reviews

RCTs, meta‐analyses and systematic reviews represent the study designs with the highest level of evidence, and therefore impose a great influence on guideline formation and clinical decisions^8^. In 2006–2007, the UK, the USA and Sweden occupied the top three positions. Ten years later, Sweden was no longer in the top three, and instead the top three were the UK, the Netherlands and the USA (*Table* 4).

**Table 4:** Top five countries ranked by number of RCTs, meta‐analyses and systematic reviews, 2006–2007 and 2016–2017

	2006–2007		2016–2017	
Rank	Country*	No. of publications	Country*	No. of publications
1	GB	36 (18·3)	GB	43 (27·4)
2	US	35 (5·8)	NL	42 (25·2)
3	SE	21 (34·4)	US	40 (4·5)
4	NL	18 (17·5)	JP	34 (8·2)
5	CN	17 (25·0)	CN	31 (21·8)

Values in parentheses are percentage of total included papers.

* Abbreviations from ISO Country Codes for Selected Countries^7^: GB, UK; US, USA; NL, the Netherlands; SE, Sweden; JP, Japan; CN, China.

### Journals ranked by total number of publications and cumulative impact factors

In 2006–2007, the five journals that published most of the papers were the *World Journal of Surgery* (15·4 per cent of all included articles), *Diseases of the Colon and Rectum* (11·7 per cent), *Annals of Surgical Oncology* (11·1 per cent), *Annals of Surgery* (10·9 per cent) and the *British Journal of Surgery* (10·1 per cent). Further details are given in *Table* 5. In 2016–2017, *Annals of Surgical Oncology* ranked first (15·2 per cent), followed by the *World Journal of Surgery* (14·3 per cent), *Obesity Surgery* (14·3 per cent), *Surgery* (11·7 per cent) and *Annals of Surgery* (9·6 per cent) (*Table* 5).

**Table 5:** All included journals ranked by total number of included articles, 2006–2007 and 2016–2017

	2006–2007			2016–2017		
Rank	Journal*	No. of included articles	CIFs	Journal*	No. of included articles	CIFs
1	*World J Surg*	347	788·7	*Ann Surg Oncol*	461	1909·0
2	*Dis Colon Rectum*	264	827·1	*World J Surg*	434	1324·6
3	*Ann Surg Oncol*	250	1057·3	*Obes Surg*	433	1743·7
4	*Ann Surg*	246	2082·1	*Surgery*	355	1349·4
5	*Br J Surg*	227	1044·7	*Ann Surg*	290	2638·1
6	*Surgery*	176	545·4	*Dis Colon Rectum*	213	840·7
7	*J Am Coll Surg*	163	494·2	*Br J Surg*	201	1216·3
8	*Obes Surg*	153	479·5	*Langenbecks Arch Surg*	179	410·1
9	*Transplantation*	151	532·6	*J Am Coll Surg*	165	820·4
10	*Dig Surg*	98	152·8	*Transplantation*	114	431·6
11	*Langenbecks Arch Surg*	96	175·6	*Dig Surg*	109	228·7
12	*J Clin Oncol*	25	343·8	*Thyroid*	37	213·5
13	*Gut*	15	124·8	*Lancet*	10	526·7
14	*Thyroid*	13	34·3	*Gut*	9	143·2
15	*N Engl J Med*	11	505·4	*Oncologist*	8	44·1
16	*Oncologist*	9	46·7	*J Clin Oncol*	7	150·1
17	*Lancet*	3	72·6	*N Engl J Med*	4	270·0
	All	2247	9307·5	All	3 029	14 260·2

* Abbreviations from the US National Library of Medicine catalogue^6^. CIF, cumulative impact factor.

In 2006–2007, most of the cumulative IFs were obtained by *Annals of Surgery* (22·4 per cent of all cumulative IFs), followed by *Annals of Surgical Oncology* (11·4 per cent), *British Journal of Surgery* (11·2 per cent), *Diseases of the Colon and Rectum* (8·9 per cent) and *World Journal of Surgery* (8·5 per cent). Ten years later *Annals of Surgery* again ranked first regarding cumulative IFs (18·5 per cent), followed by *Annals of Surgical Oncology* (13·4 per cent), *Obesity Surgery* (12·2 per cent), *Surgery* (9·5 per cent) and *World Journal of Surgery* (9·3 per cent).

Publications by general surgeons decreased from 39 papers in 2006–2007 to 21 in 2016–2017 in the high‐impact journals, *New England Journal of Medicine*, *The Lancet* and *Journal of Clinical Oncology*. Although the total number of papers included in this analysis increased from 2006–2007 to 2016–2017 by 34·8 per cent, the number of publications in these three journals decreased by 46 per cent.

## Discussion

This bibliometric analysis describes the international publication activity in general surgery over an interval of 10 years. The authors oriented their definition of general surgery according to the surgical section of the European Union of Medical Specialists, represented by the national professional and scientific associations in 26 European countries^9^, ^10^. It might be argued that the definition of general surgery is different in various countries, but at least these divisions are defined for 26 countries in Europe.

Given that English is the language used most frequently in research and most of the highest ranked journals (defined by IF) publish in English, this article focused only on English‐language publications. National journals not publishing in English, but that might have a large community of readers, were excluded. For this reason, international publication activity should not necessarily be equated with research activity. Surgeons from non‐native English‐speaking European countries, such as Germany or France, publish more articles in their own language journals (*Der Chirurg*, *Zentralblatt für Chirurgie*, *Annales de Chirurgie*, *Journal de Chirurgie*) than in international journals. This article did not include these journals, because of their low IFs. A preference to publish in non‐international journals may relate to the availability of an appropriate journal, as well as to requirements for career advancement. In Germany, for instance, there is no requirement for trainees to publish in international journals to enter an academic surgical residency programme, whereas this is often viewed as essential elsewhere in northern Europe.

This analysis suggests that, during the time period of 2006–2007, the USA was the country with the highest publication activity by far, followed by Japan and the UK. Ten years later the ranking did not change much: the USA once again occupied first place, followed by Japan and the Netherlands. This finding aligns with the only other study^2^ comparing international general surgical publication activity, albeit using a different methodology. In that study, 15 international journals containing a large amount of surgical content were analysed in the time period 2000–2005. The USA was the major contributor regarding total publications, followed by Japan, the UK, Italy and Germany^2^. The present study identified the same countries in the top five ranking for 2006–2007.

The reasons for the change in ranking between 2006–2007 and 2016–2017 cannot be elucidated from this study. Given that the highest IFs were gained by publishing randomized prospective clinical studies, the alteration in ranking must also reflect cultural acceptance of this type of study design in the respective countries. This may also reflect individual healthcare systems. Fee‐for‐service reimbursement systems as in Germany or the USA may be a disincentive to offering patients access to randomized trials, especially if there is a non‐surgical arm^11^. Differences in regulatory arrangements and funding sources may also impact on research activity and publication.

In the present study, publication activity of a country was adjusted by population size (*Table* 3). When examined in this way, in 2006–2007 Sweden, the Netherlands, Switzerland and the UK occupied the highest places, with the USA dropping to ninth place. Ten years later the Netherlands, Sweden, Switzerland and Denmark occupied the first four places. To what extent this represents higher scientific requirements, better academic training or greater proficiency in English is unclear^12^. The low‐ranking position of Germany is not surprising, given the lack of applicants for surgery residency programmes, even in university hospitals, and falling scientific/clinical requirements, as shown by a recent survey^13^.

Other studies^2^, ^14^, ^15^, ^16^, ^17^, ^18^, ^19^, ^20^, ^21^ investigating publishing trends have tended to screen journals for specific medical subjects, or for the countries of the corresponding authors without checking their affiliations. Considering these limitations, the present research is the first to address the question of which countries' surgeons contribute the most to the general surgical literature.

A limitation of this research was the choice to screen and analyse a certain selection of international journals. It might be argued, nevertheless, that these 17 leading surgical journals were likely to be representative. The selection did include various anatomical subspecialties of general surgery and a broad geographical distribution of editors. Moreover, papers from three multidisciplinary journals with high IFs were included. Although these journals did not publish many surgical articles, their high‐quality meta‐analyses and RCTs are likely to have significant impacts on surgical practice. For that reason, the UK, the Netherlands and the USA were among the top ranks in both time periods with respect to RCTs, meta‐analyses and systematic reviews.

A further difficulty of the literature search in PubMed was that for many first authors only general terms such as ‘university of’, ‘obesity centre’ and ‘others’ were stated in their affiliation information. This made it difficult to decide whether to exclude or include an article, even after group discussion. By attribution to the country of the first author, countries of the other contributing authors may have been underrepresented.
